# Interactions of Amino Group Functionalized Tetraphenylvinyl and DNA: A Label-Free “On-Off-On” Fluorescent Aptamer Sensor toward Ampicillin

**DOI:** 10.3390/bios13050504

**Published:** 2023-04-27

**Authors:** Weifu Geng, Yan Feng, Yu Chen, Xin Zhang, Haoyi Zhang, Fanfan Yang, Xiuzhong Wang

**Affiliations:** 1 College of Plant Health and Medicine, Qingdao Agricultural University, Qingdao 266109, China; gwf17854221211@163.com (W.G.);; 2 College of Chemistry and Pharmaceutical Sciences, Qingdao Agricultural University, Qingdao 266109, China

**Keywords:** aggregation-induced emission, DNA, label-free, biosensor, antibiotic

## Abstract

As a type of aggregation-induced emission (AIE) fluorescent probe, tetraphenylvinyl (TPE) or its derivatives are widely used in chemical imaging, biosensing and medical diagnosis. However, most studies have focused on molecular modification and functionalization of AIE to enhance the fluorescence emission intensity. There are few studies on the interaction between aggregation-induced emission luminogens (AIEgens) and nucleic acids, which was investigated in this paper. Experimental results showed the formation of a complex of AIE/DNA, leading to the quenching of the fluorescence of AIE molecules. Fluorescent test experiments with different temperatures proved that the quenching type was static quenching. The quenching constants, binding constants and thermodynamic parameters demonstrated that electrostatic and hydrophobic interactions promoted the binding process. Then, a label-free “on-off-on” fluorescent aptamer sensor for the detection of ampicillin (AMP) was constructed based on the interaction between the AIE probe and the aptamer of AMP. Linear range of the sensor is 0.2–10 nM with a limit of detection 0.06 nM. This fluorescent sensor was applied to detect AMP in real samples.

## 1. Introduction

Twenty years ago, Tang’s research group found a new class of organic molecules which had almost no emission in dilute solutions, but strong fluorescence emission in an aggregate or solid state, which they termed aggregation-induced emission luminogens (AIEgens) [1]. AIE molecules could avoid concentration quenching or aggregation-caused quenching (ACQ) of the conventional fluorophores [2]. Therefore, AIEgens molecules have been used to construct chemicals and biosensors [3], bioimaging probes, and have been widely applied in various fields over the past twenty years [4]. As typical AIE dyes, tetraphenylethene (TPE) and its derivatives have been widely used to construct fluorescent sensors and biological probes [5,6]. Li and coworkers [7] developed a fluorescent aptasensor for the detection of exosome tumor-associated proteins, combining aptamers, tertiary amine-containing tetraphenylethene, and graphene oxide (GO) as recognition units, fluorescent probe, and the quencher, respectively. Numerous AIE molecules could bind to the aptamer and form aggregates rapidly, leading to an amplified fluorescence intensity. Zhu et al. [8] constructed a label-free and turn-on AIE-based fluorescence aptamer sensor for the detection of mycotoxin. Hu [9] prepared AIE-Red and AIE-Green fluorescence probes, using the same TPE cores functionalized with the positively charged morpholine groups or vinyl pyridinium. With only one excitation laser, this strategy can facilitate the multicolor monitoring of mitochondria and lysosomes, respectively. Xu et al. [10] reported a TPE-based fluorescent probe by decorating carboxylate coordination groups on TPE for the highly selective and sensitive detection of nitrofuran in aqueous solution. Because TPE molecules are insoluble in water, most of the reports focused on functional modification of the TPE core skeleton to enhance its practicability which led to the complex molecular structures of TPE derivatives, with complex synthesis steps and a high cost [11,12,13]. Therefore, it is necessary to develop new sensitive and accurate biosensors with simple molecule probes without complex functionalization in the TPE-based fluorescence sensing systems.

Aptamer is a single-stranded DNA (ssDNA), RNA, or modified nucleic acid isolated through SELEX technology, and can specifically bind to corresponding targets, for example, small organic molecules, viruses or proteins with high affinity and selectivity [14,15]. Aptamer-based sensors (Aptasensor) have been widely reported and developed in recent years [16,17,18,19]. AIE molecules, generally having a positive charge, would tend to bind to negatively charged aptamers, and the fluorescence emission varies with the change of the molecular aggregation state [8,20]. Zhang et al. [21] developed a turn-on fluorescent sensor for the detection of chloramphenicol in which an AIE 9,10-distyrylanthracene derivative with short alkyl chains and GO function as the fluorescent probe and the fluorescence quencher, respectively. However, the interaction between AIEgens and DNA strands has been rarely reported. To illustrate the binding mechanism of AIEgens to DNA, the interactions between the amino group functionalized TPE (TPE-Am) and single-stranded DNA were studied in this paper. The binding types, constants and types of force were investigated. Experimental results showed that the binding modes between AIE molecule and DNA were intercalation and groove binding. Fluorescent test experiments under different temperatures proved that the quenching type was a static quenching. The quenching constants, binding constants and thermodynamic parameters demonstrated that electrostatic and hydrophobic interactions promoted the binding process.

Antibiotics are generally used to treat infections caused by pathogenic bacteria or fungi in healthcare. However, the overuse of antibiotics may lead to food and environmental pollution [22,23,24]. Ampicillin (AMP) is widely used to manage and treat certain bacterial infections. Therefore, it is very important to detect AMP residues in natural environments and foodstuffs for human health protection. Up to now, various detection methods such as chromatography, microbiological assay, electrochemical and photochemical sensors have been developed to detect antibiotics [25,26,27,28,29,30]. Chromatography has great advantages on selectivity; however, it requires professional instruments and cumbersome pretreatment. Sensitivity and specificity of microbiological assay for the detection of antibiotics is not enough. In spite of high detection sensitivity, electrochemical sensors are prone to contamination and have poor reproducibility. Compared with other detection methods, photochemical sensors, especially fluorescent sensors for the detection of antibiotics, have received more and more attention in recent years. However, most of the detection methods need fluorescent labeling which are time-consuming and costly. Therefore, it is urgent to develop a simple, fast and inexpensive detection method for AMP.

Based on the aforementioned developments, in this paper we developed a simple, label-free fluorescent aptasensor, with high specificity and sensitivity, to detect AMP using the AIE probe based on interaction between AIEgens and the aptamer of AMP.

## 2. Materials and Methods

### 2.1. Reagents and Materials

The amino group functionalized TPE (TPE-Am) was donated by professor Lei Han (Qingdao Agricultural University). Ampicillin (AMP) was bought from Macklin (Shanghai, China). Other antibiotic and pesticides, including penicillin (PEN), chloramphenicol (CHL), roxithromycin (ROX), tetracycline (TET), imidacloprid (IMI), and methidathion (MET) were bought from Aladdin Chemical Co., Ltd. (Shanghai, China). Dimethyl sulfoxide was bought from Kangde (Laiyang, China). Na_2_HPO_4_, KH_2_PO_4_, NaCl and KCl were purchased from Tianjin Basf Chemical Co., Ltd. (Tianjin, China). The DNA sequences 5′-GCGGGCGGTTGTATAGCGG-3′ (aptamer for AMP) was obtained by Sangon Biotech (Shanghai, China). All of the DNA was dispersed in 10 mM phosphate-buffered solution (PBS) and stored at −18 °C before use. All chemicals and solvents used in the experiment were of analytical grade. Ultrapure water used in the experiments was obtained from a Milli-Q water purification system (Millipore Corp., Burlington, MA, USA).

### 2.2. Measurement and Apparatus

Fluorescence tests were performed on a Gangdong technology F-380 fluorescence spectrometer (Tianjin, China) equipped with 1.0 cm micro quartz cells. The excitation wavelength was set at 340 nm and the fluorescence spectra were collected from 400 to 625 nm. A fluorescence intensity of 490 nm was used to evaluate the performance of the sensing system. The pH of solutions was adjusted by a PB-10 digital pH meter (Sartorius, Shanghai, China). The ultraviolet-visible absorption spectra were collected at room temperature on a U3900 spectrophotometer (Hitachi, Tokyo, Japan) equipped with 1.0 cm micro quartz cells.

### 2.3. Principles of Fluorescence Quenching

The fluorescence quenching process is usually described according to the Stern–Volmer Equation (1) [31]:

(1) $$F_{0}/F=1+k_{q}\tau_{0}[Q]=1+K_{sv}[Q]$$

where *F* and *F*_0_ represents the steady state fluorescence emission intensities with and without quencher, respectively, *k_q_* is rate constant of the dynamic fluorescence quenching process, *τ*_0_ is the fluorescence lifetime without quencher in the system, [*Q*] is the concentration of the quencher, and *K_SV_* is the quenching constant. Hence, *K_SV_* can be determined according to Equation (1) using a linear regression plot of *F*_0_*/F* against [*Q*].

### 2.4. Fluorescence Detection Procedures for AMP 

A volume of 10 μL of AMP aptamer (10 μM) and 2 μL TPE-Am (100 μM) were incubated for 30 min in 99% PBS (PBS:DMSO, *v*:*v*) at 25 °C. Then, a different concentration of AMP was introduced into the above solution and the final solution was fixed to 200 μL. After 45 min incubation at 25 °C, the fluorescence intensity of the reaction solution was measured. As control experiments, penicillin (PEN), chloramphenicol (CHL), roxithromycin (ROX), tetracycline (TET), imidacloprid (IMI) and methidathion (MET) were instead incubated with AMP, respectively.

For AMP content detection in real samples, water samples were collected from the Hongzi river located in the Chengyang District (Qingdao, China) and were pretreated according to the literature [32].

## 3. Results and Discussion

### 3.1. Fluorescence Spectroscopy of TPE-Am

**Figure 1:**
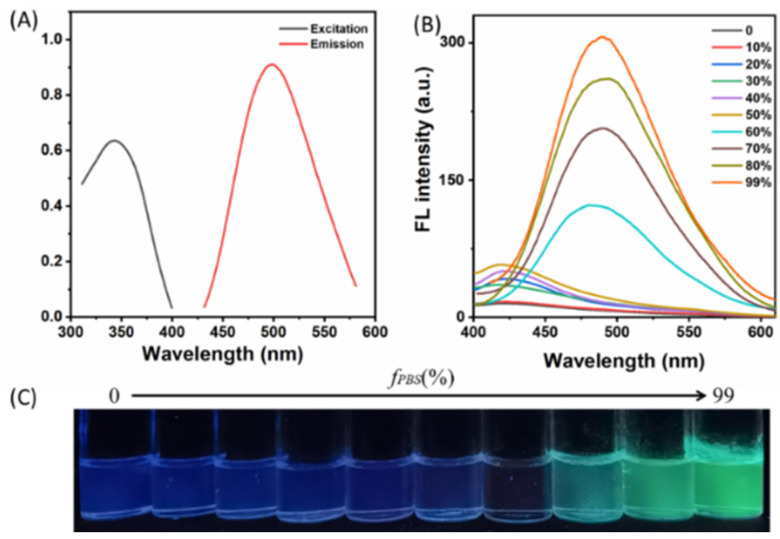
Fluorescence spectroscopy of TPE-Am. (**A**) Normalized excitation and emission profile of TPE-Am in PBS/DMSO mixture (*f_PBS_* = 99%); (**B**) the AIE characteristics of TPE-Am in DMSO and PBS/DMSO mixture; (**C**) photograph of TPE-Am in DMSO and PBS/DMSO mixtures with different PBS fractions (*f_PBS_*).

The excitation and emission spectra of TPE-Am in 99% PBS (vs. DMSO) were firstly tested. Figure 1A shows that the maximum excitation and emission wavelengths of the AIE molecule are 340 nm and 490 nm, respectively, which is consistent with the literature [33]. Next, aggregation-induced emission characteristics of TPE-Am was investigated. According to the molecular structure of TPE-Am (Figure S1, in the Supplementary Materials), as is well known, it is an insoluble and commercially available molecule. We investigated AIE characteristics of TPE-Am by investigating photoluminescence behaviors in the DMSO and PBS/DMSO mixtures. As shown in Figure 1B, TPE-Am (1 μM) almost has no emission in diluted DMSO solution, but the fluorescence intensity of TPE-Am increases with increasing PBS fraction (*f_PBS_*) from 0 to 99% (vs. DMSO). A photograph of TPE-Am in the DMSO and PBS/DMSO mixtures with different PBS fractions (*f_PBS_*) is shown in Figure 1C. These phenomena indicate that TPE-Am enhanced emission in PBS/DMSO mixtures is derived from its aggregation in poor solvents.

### 3.2. Interaction of TPE-Am and DNA

**Figure 2:**
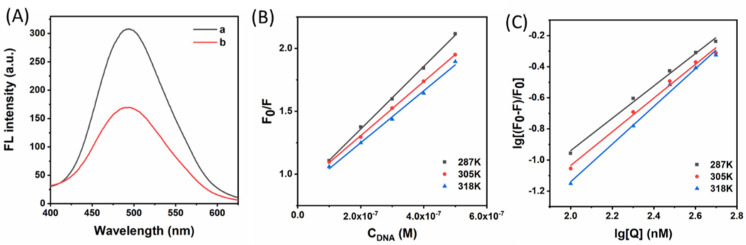
Interaction of TPE-Am and DNA strands (**A**). FL intensity of the system with different conditions: (a) TPE-Am and (b) TPE-Am + DNA. (**B**) The fluorescence quenching tests at different temperature. (**C**) Stern–Volmer plots for the TPE-Am/DNA system with different temperatures.

Binding modes between TPE-Am and DNA were examined by fluorescence spectroscopy. The fluorescent emission spectra of TPE-Am and TPE-Am/DNA strands (the aptamer of AMP) are illustrated in Figure 2A. The fluorescence intensity significantly decreases (curve b) in the presence of 500 nM DNA strands compared with TPE-Am alone (curve a).

#### 3.2.1. Quenching Mechanism of Fluorescence

Dynamic or static quenching mechanisms could be judged from constants *K_q_* and *K_sv_* at different temperatures. Because of its dependance on diffusion effects, *K_q_* and *K_sv_* were larger with increasing temperature. However, non-fluorescing complexes were produced during static quenching process, leading to decreasing static quenching constants with increasing temperature [34,35].

Next, the fluorescence quenching tests were carried out at a temperature of 287, 305 and 318 K between TPE-Am and the aptamer of AMP, respectively, which are shown in Figure 2B. The *K_SV_* and *K_q_* values were calculated according to Equation (1) and are exhibited in Table 1. The experimental results demonstrated a classical static quenching process for TPE-Am binding to DNA strands, as the values of *K_q_* were much larger than 2 × 10^10^ L·mol^−1^s^−^^1^ which was the maximum scattering collision quenching constant. The *K_sv_* values decreased with increasing temperature; furthermore, the dynamic quenching constant was generally less than 100 L·mol^−1^.

#### 3.2.2. Binding Constants

For the static quenching process, the binding constant (*K_b_*) and the number of TPE-Am-bound sites (*n*) per DNA strand could be determined from Equation (2).

(2) $$\mathrm{lg}\frac{F_{0}-F}{F}=\mathrm{lg}K_{b}+n\mathrm{lg}[Q]$$

Parameters *F*_0_, *F* and [*Q*] express the same meanings as in Equation (1). *K_b_* and *n* could be obtained from the intercept and slope by plotting lg [*(F*_0_ *− F)*/*F*] against lg [Q] (intercept = lg*K_b_*, slope = *n*) (Figure 2C), and the values of *K_b_* and *n* are listed in Table 2. The value of *K_b_* decreased with increasing temperature according to Table 2 which was in accordance with *K*_sv_, showing moderate binding between TPE-Am and DNA, and that TPE-Am/DNA complex might be formed [36]. The values of *n* are equal to approximately 1, indicating that there was one class of binding sites for TPE-Am in DNA.

#### 3.2.3. Thermodynamic Constants

It is well known that the four major interaction forces between small organic molecules and biomacromolecules are electrostatic interactions, hydrogen bonds, hydrophobic force, and van der Waals force, respectively. The enthalpy change (Δ*H*) of the interaction usually remains stable if the temperature does not change much. For small molecules binding to DNA, the interaction types and modes can be estimated by thermodynamic parameters, including free energy change (Δ*G*), enthalpy change (Δ*H*), and entropy change (Δ*S*), according to Equations (3) and (4):

(3) $$\ln K=-\frac{\Delta H}{RT}+\frac{\Delta S}{R}$$

(4) ΔG=−RTlnK=ΔH−TΔS

where *K* is the binding constant (*K_b_*) at 287, 305 and 318 K, respectively, and *R* is the gas constant which is 8.314 J mol^−1^·K^−1^. The corresponding values were obtained from Equations (3) and (4) and listed in Table 2. The electrostatic and hydrophobic interactions were the main interaction force between TPE-Am and DNA, judging from the negative Δ*H* and positive Δ*S* value [37].

### 3.3. Fluorescence Assay for AMP

**Scheme 1:**
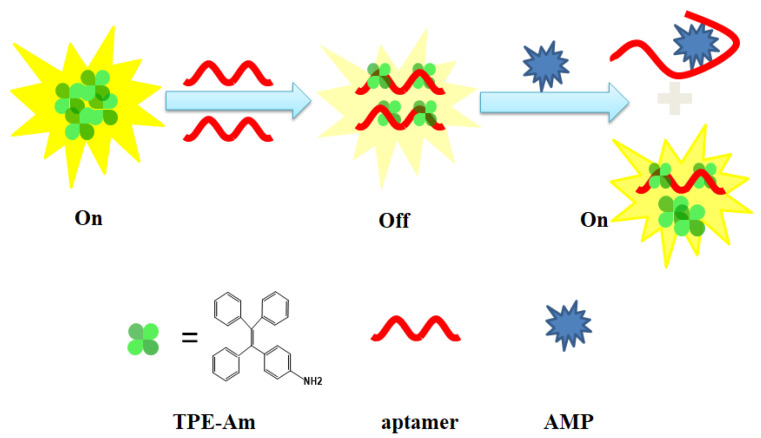
Sensing mechanism of the proposed aptasensor for detection of AMP.

A facile, sensitive and label-free “on-off-on” fluorescent aptamer sensor for AMP detection was successfully developed based on the interaction between the TPE-Am probe and aptamer of AMP which is illustrated in Scheme 1. There was strong fluorescence in 99% PBS (PBS:DMSO, *v*:*v*) because of the hydrophobicity of TPE-Am. In the presence of the aptamer of AMP (Apt), the weakened fluorescence intensities of the system was the result of the hydrophobic interaction between the TPE-Am probe and DNA (from on to off). However, the fluorescence recovered once the target AMP molecules were introduced into the solution (from off to on). As is well known, the force between the target and the aptamer is much larger than the hydrogen bonds and hydrophobic interactions. Fluorescence recovery came from the release of the TPE-Am probe because of the formation of an Aptamer-AMP complex. Therefore, the detection of AMP can be realized by monitoring the changes of the fluorescence intensities using this on-off-on fluorescent aptamer sensor.

#### 3.3.1. Characterization of TPE-Am/Aptamer

**Figure 3:**
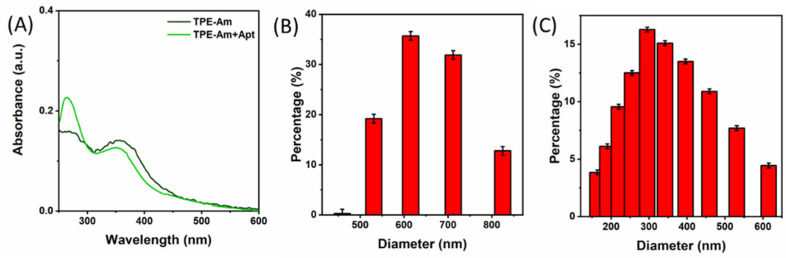
Characterization of TPE-Am/Aptamer. (**A**) UV absorption spectrum of TPE-Am and TPE-Am/Aptamer. (**B**) Size distribution of TPE-Am. (**C**) Size distribution of TPE-Am/Aptamer.

UV-vis absorption spectra were used to verify the complex of TPE-Am and Aptamer. It could be seen from Figure 3A that both the TPE-Am and the TPE-Am/Aptamer complex had the same absorption bands at the wavelength range of 300–360 nm which is the representative absorption peak of a tetraphenylethylene structure [33]. Compared with the TPE-Am, the absorption band of the TPE-Am/Apt complex were in the wavelength range of 260–280 nm which is the same as that of the typical structural features of DNA.

Dynamic light scattering (DLS) was used to test the size distribution of TPE-Am in 99% PBS (PBS:DMSO, *v*:*v*). The mean particle size changed from 610 nm (Figure 3B) to 300 nm (Figure 3C), and the reason is that the formed TPE-Am aggregates tend to disperse upon the formation of the TPE-Am/Aptamer complex. These results clearly indicated that the TPE-Am probe was successfully grafted on the aptamer of AMP.

#### 3.3.2. Feasibility of the Fluorescence Aptamer Sensor

**Figure 4:**
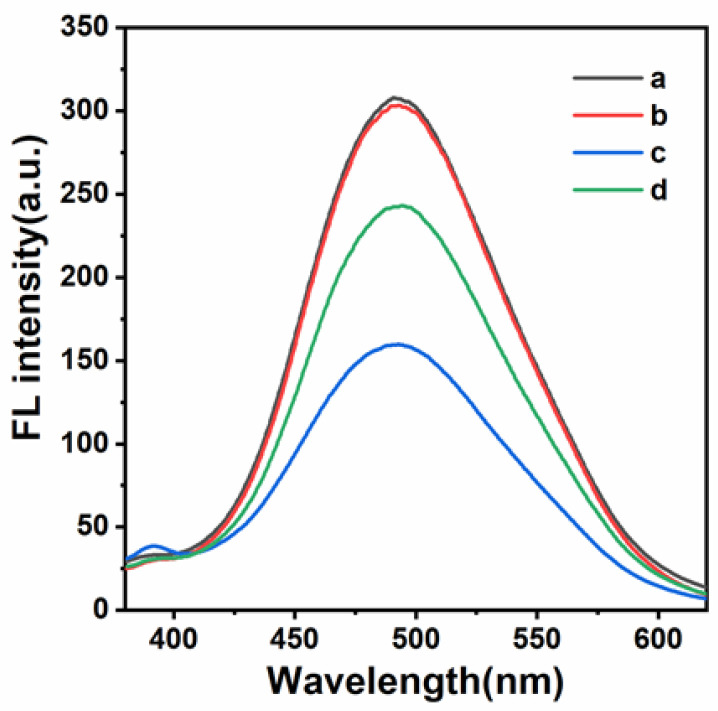
Fluorescence emission under different conditions: (a) TPE-Am, (b) TPE-Am + AMP, (c) TPE-Am + Apt, and (d) TPE-Am + Apt + AMP.

Figure 4 shows the fluorescent emission spectra of TPE-Am (1.0 μM) under different conditions. The fluorescence intensity was largest with TPE-Am alone in 99% PBS (PBS:DMSO, *v*:*v*) (curve a). There was little change in the fluorescence intensities when the AMP targets were added (curve b) compared with TPE-Am alone. Fluorescence intensities significantly decreased in the presence of the aptamer of AMP (curve c). Nevertheless, fluorescence obviously recovered when the AMP targets were added into the solution because of the formation of the Aptamer-AMP complex accompanied with the release of the TPE-Am probe (curve d). Therefore, AMP content could be detected by monitoring the changes of the fluorescence intensities using this on-off-on fluorescent aptamer sensor.

#### 3.3.3. Optimization of the Experimental Conditions

In order to obtain the optimal performance of the proposed fluorescent sensor for detection of AMP, the experimental conditions, including the concentrations, response times, and pH of the buffer solution, were optimized, respectively. As shown in Figures S2–S5, it was observed that the optimal concentration of aptamer, incubation time for TPE-Am/Aptamer, TPE-Am/Aptamer/AMP and pH of the solution were 500 nM, 30 min, 45 min, and 7.4, respectively.

#### 3.3.4. Analytical Performance of Fluorescent Aptasensor

**Figure 5:**
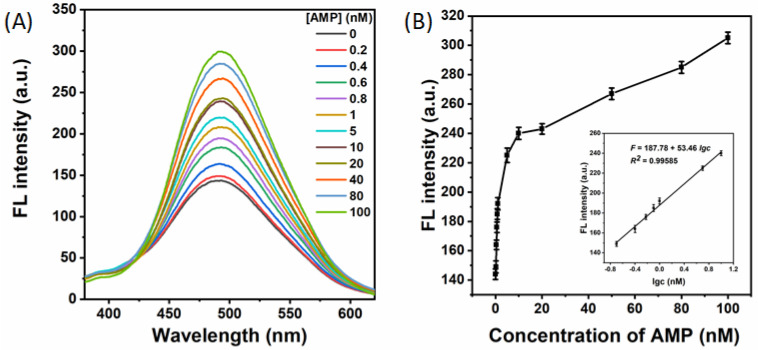
(**A**) Fluorescence responses of the aptasensor to various concentrations of AMP (0, 0.2, 0.4, 0.6, 0.8, 1, 5, 10, 20, 50, 80 and 100 nM). (**B**) The relationship between fluorescence intensity (λem = 493 nm) and the concentration of AMP. Inset: the linear relationship between fluorescence intensity and the logarithm value of AMP concentration.

The fluorescent aptasensor was evaluated by monitoring the fluorescence change under different concentrations of AMP. As shown in Figure 5A, the fluorescence intensity increased gradually with increasing AMP concentration. A good linear relationship between the fluorescence intensity and the AMP concentration was found, ranging from 0.2 to 10 nM, which is shown in the inset of Figure 5B. The linear regression equation was *F* = 53.46 *lgc* + 187.78 with a correlation coefficient of 0.9959 (*F*: fluorescence intensity, c: AMP concentration (nM)). The limit of detection was estimated to be 0.06 nM (based on 3σ) which was lower than those of the previously reported assay methods (Table S1).

#### 3.3.5. Selectivity of the Sensor for Detection of AMP

**Figure 6:**
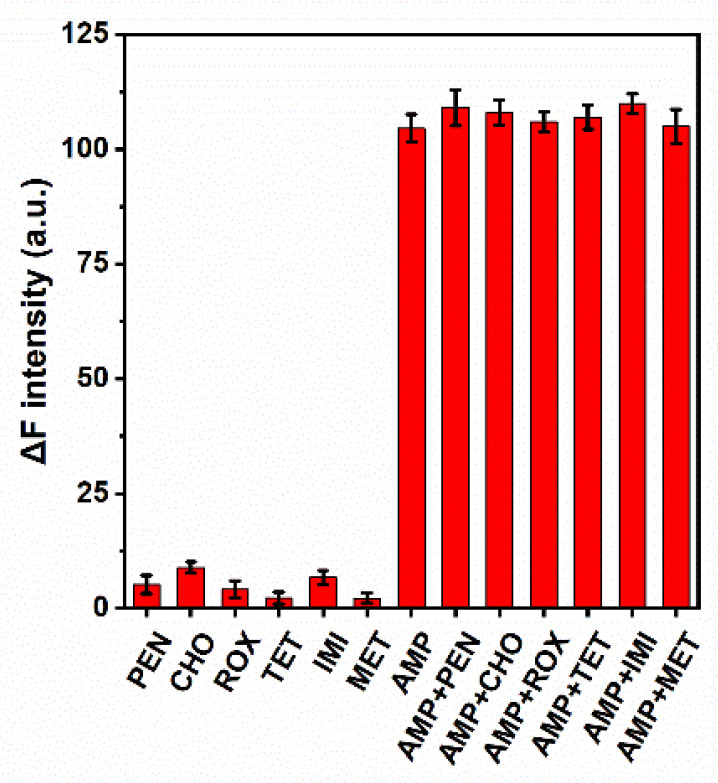
The selectivity of the aptasensor responds to AMP in the presence of PEN, CHO, ROX, TET, IMI, and MET. The concentration of AMP was 10 nM and that of the other disruptors was 100 nM. The error bars represent the standard deviation of three repeated measurements.

The selectivity of the aptasensor for AMP was investigated. The controlled experiments were carried out by selecting several common interfering substances such as PEN, CHL, ROX, TET, IMI, and MET under the same experimental conditions. As shown in Figure 6, compared with the background signal, the fluorescence intensities were barely changed in the presence of interfering substances; obvious fluorescence intensity change was obtained only in the presence of AMP. These data indicated that this aptasensor exhibited a good performance in discriminating AMP from other interfering antibiotics and pesticides. Therefore, it has good potential to be applied in real complex samples.

#### 3.3.6. AMP Content in Real Samples

To investigate the applicability of the sensing strategy in real samples, it was applied to detect AMP residues in natural water. The experimental results demonstrated that the level of AMP residues in the water samples was below the limit of detection. Next, samples were tested by adding different amounts of AMP (0.2, 0.6 and 1 nM) in the river water samples, with three repeated measurements, and recovery from 99.6% to 102.85% was obtained (Table S2). These results indicated that the sensing strategy has good anti-interference capabilities and therefore shows strong potential application in food safety fields.

## 4. Conclusions

In summary, the interaction between the amino group functionalized TPE molecule and nucleic acids was investigated in detail by fluorescence and UV–vis absorption spectroscopy in this paper. Experimental results showed that the amino group functionalized TPE could combine with DNA by intercalation and groove binding force. Binding and thermodynamic constants demonstrated that the fluorescence quenching mechanism of the TPE molecule by DNA is a static quenching procedure and that the electrostatic and hydrophobic interactions were the main driving force between TPE-Am and DNA. Based on the above, a sensitive and label-free “on-off-on” fluorescent aptamer sensor for AMP detection was developed. The sensor has a linear range of 0.2–10 nM with a limit of detection of 0.06 nM. It was used to detect the AMP content in real samples.

## Figures and Tables

**Table 1:** Stern–Volmer quenching constants of TPE-Am binding to DNA.

*T* (K)	*K_sv_* (L·mol^−1^)	*K_q_* (L·mol^−1^·s^−1^)	*R* ^1^
287	2.488 × 10^6^	2.488 × 10^14^	0.9986
305	2.149 × 10^6^	2.149 × 10^14^	0.9995
318	2.065 × 10^6^	2.065 × 10^14^	0.9949

^1^ *R* is the correlation coefficient.

**Table 2:** The binding parameters and relevant thermodynamic parameters for the TPE-Am―DNA system.

*T* (K)	*K_b_* (/10^5^ L/mol)	*n*	*R*	Δ*H* (kJ/mol)	Δ*S* (J/mol)	Δ*G* (kJ/mol)
287	9.59	1.03	0.9929	−29.46	12.59	−33.07
305	6.26	1.08	0.9902			−33.3
318	2.75	1.21	0.9902			−33.46

## Data Availability

The data that supports the findings of this study are available in the supplementary material of this article.

## Supplementary Materials

The following supporting information can be downloaded at: https://www.mdpi.com/article/10.3390/bios13050504/s1, Figure S1: Molecular structure of TPE-Am; Figure S2: The effect of aptamer concentration; Figure S3: The effect of incubation time for the TPE-Am/Aptamer; Figure S4: The effect of incubation time for the TPE-Am/Aptamer/AMP; Figure S5: The effect of pH to the response of the sensing strategy; Figure S6: Repeatability and stability test of the aptasensor. The concentration of AMP is 10 nM; Table S1: Comparison of various detection methods for ampicillin; Table S2: The recovery and RSD for the detection of AMP in river water samples.
