# Supplementary material for: Interactions of Amino Group Functionalized Tetraphenylvinyl and DNA: A Label-Free “On-Off-On” Fluorescent Aptamer Sensor toward Ampicillin
Source: Biosensors (Basel). 2023 Apr 27;13(5):504. doi: 10.3390/bios13050504 (PMC10216731; doi:10.3390/bios13050504)
Supplement: Supplementary file 1 [file biosensors-13-00504-s001.zip › biosensors-2303061-supplementary.pdf]

Supporting Information

Interactions of Amino Group Functionalized Tetraphenylvinyl and DNA: A Label-Free “On-Off-On” Fluorescent Aptamer Sensor toward Ampicillin

Weifu Geng [1,†], Yan Feng [2,†], Yu Chen [2], Xin Zhang [1], Haoyi Zhang [2], Fanfan Yang [2] and Xiuzhong Wang [1,2,*]

[1] College of Plant Health and Medicine, Qingdao Agricultural University, Qingdao 266109, China; gwf17854221211@163.com (W.G.)
[2] College of Chemistry and Pharmaceutical Sciences, Qingdao Agricultural University, Qingdao 266109, China
* Correspondence: xzwang@qau.edu.cn; Tel.: +86-532-58957442
† These authors contributed equally to this work.

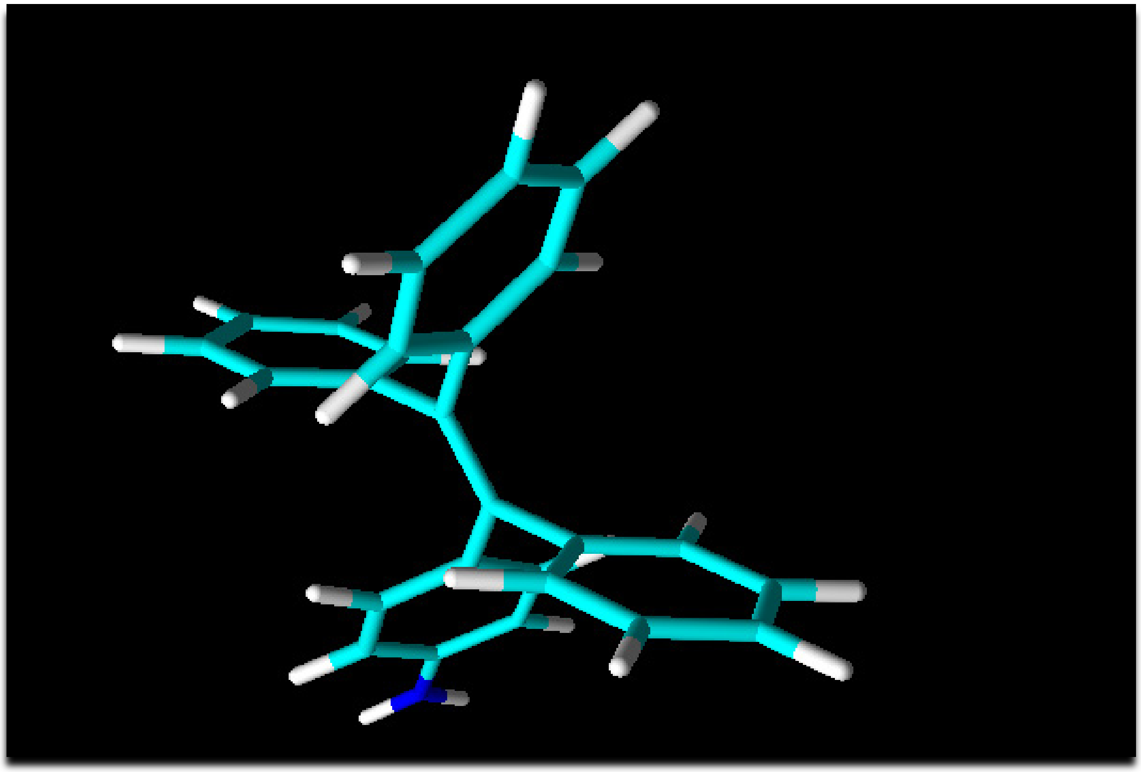

Figure S1. Molecule structure of TPE-Am

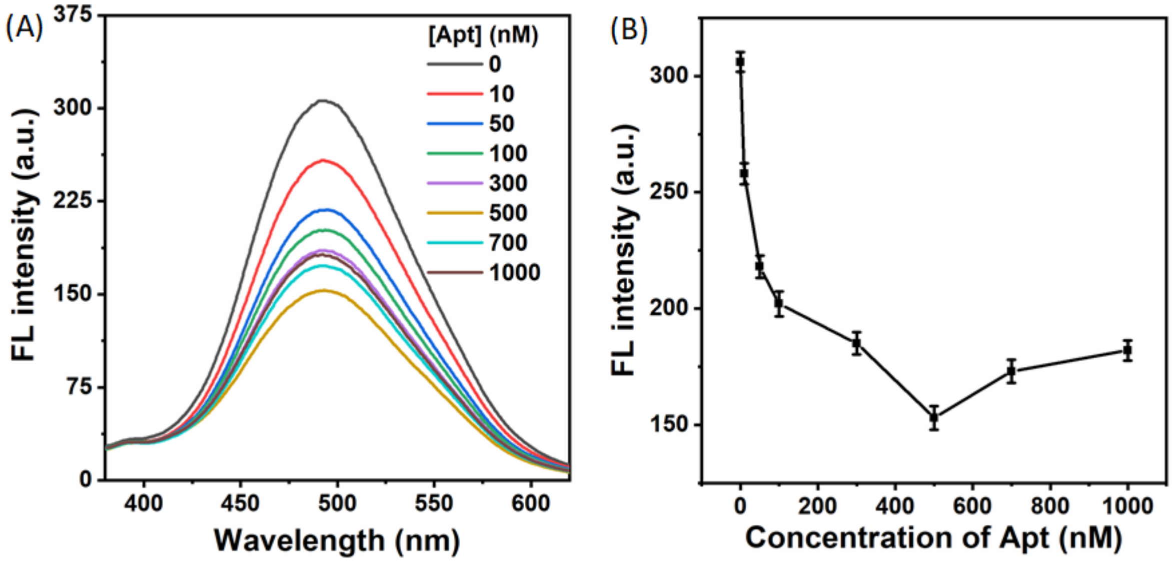

Figure S2. The effect of aptamer concentration

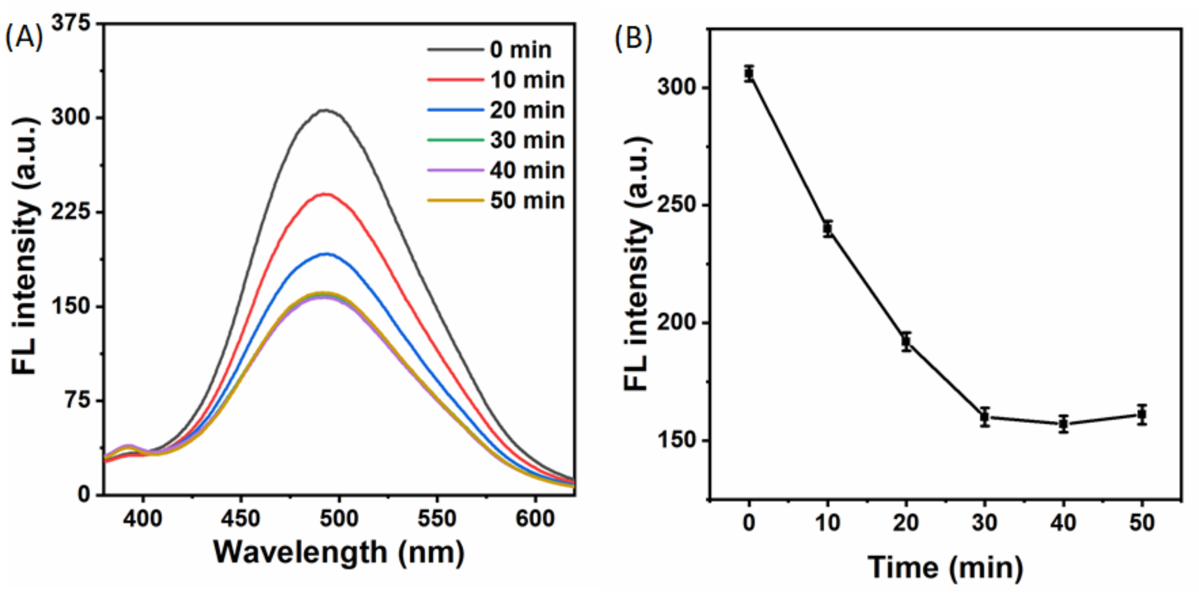

Figure S3. The effect of incubation time for TPE-Am/Aptamer

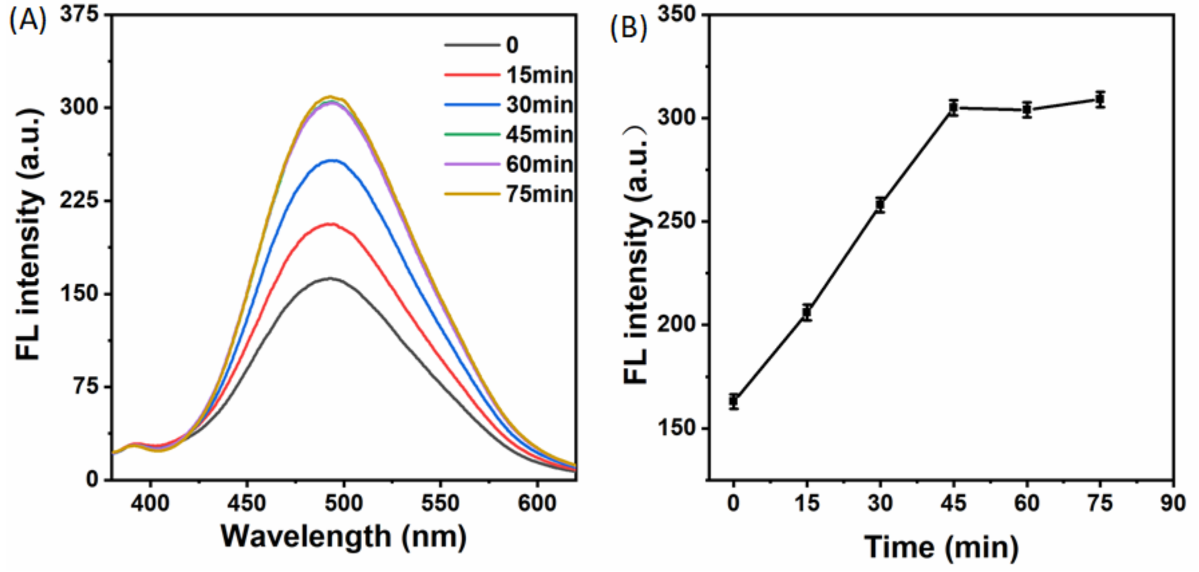

Figure S4. The effect of incubation time for TPE-Am/Aptamer/AMP

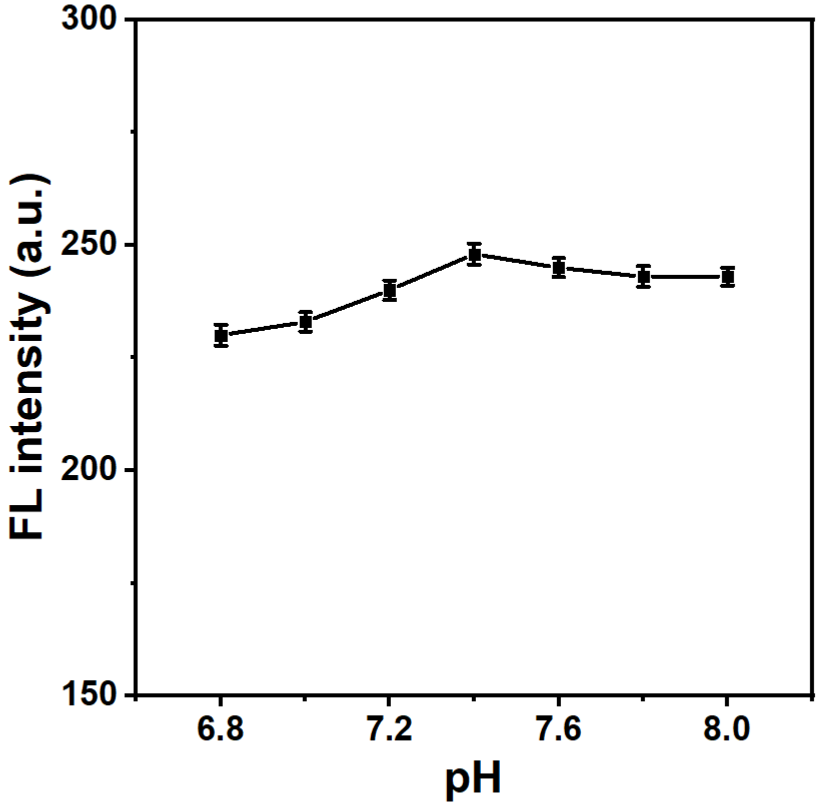

Figure S5. The effect of pH to the response of sensing strategy

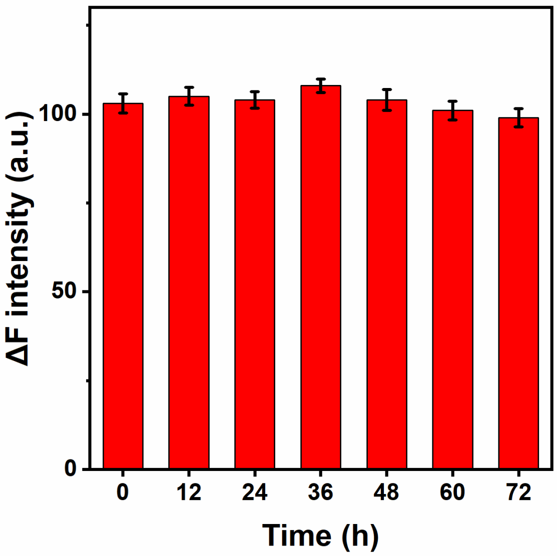

Figure S6. Repeatability and stability test of this aptasensor. The concentration of AMP is 10 nM.

Table S1. Comparation of vary detection methods for ampicillin.

Analysis methods	LOD (μM)	Linear range (μM)	References
Fluorescence method	0.035	0.75–40	[5]
Fluorescence method	0.700	0–60	[6]
Electrochemical aptasensor	0.0330	0.1–1,000	[7]
Fluorescent aptasensor	0.0260	0.1–100	[8]
Photochemical aptasensor	9.3×10^{-5}	1×10^{-4}–0.2	[9]
Chemiluminescence	0.261	0.4–4.0	[10]
Chemiluminescence	0.014	0.020–1.0	[12]
Electrochemical aptasensor	1.0000	5–5,000	[13]
Surface plasmon resonance	1.000	2.5–1,000	[14]
Fluorescence aptasensor	6×10^{-5}	$2\times10^{-4} – 0.01$	This study

Table S2. The recovery and RSD for the detection of AMP in river water samples.

No.	Measured (nM)[a]	Added (nM)	Found (nM)	Mean recovery (%)	RSD[b] (%, n=3)
1	ND	0.20	0.206	102.85	4.84
2	ND	0.60	0.598	99.67	3.34
3	ND	1.00	0.990	99.60	4.41

[a] "ND" means that the concentration of AMP in river water samples is too low to be detected.

[b] RSD (relative standard deviation) was calculated from three separate experiments.
